# Evaluating the Potential Inhibition of PP2A by Nodularin-R Disinfection By-Products: Effect and Mechanism

**DOI:** 10.3390/toxins17100484

**Published:** 2025-09-26

**Authors:** Mengchen Li, Chunyu Fu, Qiannan Shi, Shaocong Yang, Wansong Zong

**Affiliations:** College of Geography and Environment, Shandong Normal University, 88# East Wenhua Road, Jinan 250014, China; mengchenli007@163.com (M.L.);

**Keywords:** nodularin-R, disinfection by-products, protein phosphatase 2A, homology modeling, molecular docking

## Abstract

The secondary contamination of nodularin disinfection by-products (NOD-DBPs) is a problem worthy of attention. In this study, prototypical NOD-R-DBPs were prepared, and their toxicity was assessed using conventional protein phosphatase (PPs) inhibition assay, confirming that structural changes in “Adda^3^” during chlorination are key factors leading to a significant reduction in NOD-R toxicity. However, some NOD-R-DBPs still exhibit certain levels of toxicity (2.8–81% of NOD-R). To elucidate the mechanism underlying the potential inhibitory effect of NOD-R-DBPs on protein phosphatase 2A (PP2A), molecular simulations were employed to establish interaction models between prototypical NOD-R-DBPs and PP2A using homology modeling strategies, and molecular docking was used to obtain candidate interaction parameters between prototypical NOD-R-DBPs and PP2A. Structural changes in “Adda^3^” weakened the hydrogen bonds “Adda^3^”Asn_117_ and “Adda^3^”His_118_. Subsequently, the disruption of “Adda^3^” altered key interactions between NOD-R-DBPs and PP2A (hydrogen bond Mdhb^5^ ← Arg_89_, ionic bond Glu^4^-Arg_89_, metal bond His_241_-Mn_1_^2+^, etc.). The changes in these interactions further altered the interactions between conserved amino acids and the catalytic center Mn^2+^ (ionic bond Asp_57_-Mn_2_^2+^), thereby increasing Mn^2+^ exposure. Meanwhile, the retained interactions promoted the binding of -PO_4_ with the conserved amino acids His_118_ and Arg_89_. Prototypical NOD-R-DBPs retained the aforementioned key interactions and thus exhibit potential inhibitory effects on PP2A. The varying degrees of damage to the Adda^3^ structure led to significant differences in the inhibitory effects of different NOD-R-DBPs on PP2A.

## 1. Introduction

The increasing frequency of algal blooms is driving heightened concern regarding the toxicity of *Nodularia spumigena* to both animals and humans [1]. In natural settings, numerous wild and domesticated animals succumb to the effects of freshwater contaminated with the hepatotoxin nodularin [2]. Nodularins (NODs) are potent hepatotoxins produced by *Nodularia spumigena*, which are mainly found in brackish or semi-saline waters and estuarine environments [3,4]. NODs are structurally similar to microcystins (MCs) and are cyclic pentapeptides consisting of five variable amino acids, with the chemical structure of cyclo-D-MeAsp^1^-L-Arg^2^-Adda^3^-D-Glu^4^-Mdhb^5^, where Mdhb^5^ is 2-(methylamino)-2-dehydrobutanoic acid, MeAsp^1^ is D-erythro-β-methylaspartic acid, and Adda^3^ is the unique β-amino acid: 3-amino-9-methoxy-2,6,8-trimethyl-10-phenyldeca-4,6-dienoic acid [4,5,6]. In addition, Arg^2^ is arginine, and Glu^4^ is glutamic acid. Mutations occurring within the Adda^3^ residue reduce or eliminate the toxicity of the compound; the variable amino acid at position 2 can be substituted to generate other substances, esterification of the free carboxyl group of the D-Glu^4^ residue eliminates the toxicity, and the position 1 variation has less impact [7]. NODs and MCs are both cyclic peptides with similar amino acid compositions. To date, studies on them have been very limited, and since NODs have a similar chemical structure to MCs, their toxicity can be estimated on the basis of MCs [8]. At subacute doses, NODs, like MCs, are considered as initiators and promoters of liver tumors [9].

The hepatotoxicity and carcinogenicity of NODs are related to their inhibitory effects on eukaryotic protein phosphatase (PP), catalytic subunit 1 (PP1), and 2A(PP2A), and the inhibitory effect on PP2A is greater than that on PP1 [7]. Acute toxicity of NODs mainly leads to liver dysfunction and structural destruction of hepatocytes [10]. The mechanism of toxic action is the presence of hydrophobic C_20_ β-amino acid Adda^3^ in NODs, which blocks PP activity by interacting with the hydrophobic groove, forming a cage-like structure that rapidly wraps the Adda^3^ side chain and prevents substrate access to the active site [11,12]. Inhibition of phosphatase leads to cellular hyperphosphorylation, disruption of cellular metabolism, and cell cycle control, and consequently organ failure [13]. Mdhb^5^ binds to the Cys_273_ of PP2A in a manner similar to the Mdha^5^ residue in microcystin [14]. However NODs differ from MCs in that inhibition of phosphatase is characterized by non-covalent binding [5], which may account for its additional oncogenic properties.

In order to effectively reduce or eliminate the environmental risk of NODs, disinfection techniques have been widely used to control NOD contamination of water bodies [15]. For example, chlorination disinfection processes are widely used in drinking water treatment. Disinfectants can break down NODs into less or non-toxic substances by disrupting their key structures. However, these treatment processes can produce various primary NOD-related disinfection by-products (NOD-DBPs). Disinfection by-products (DBPs) are a type of secondary pollutant generated by the reaction of disinfectants with organic or inorganic precursors during drinking water disinfection. They have carcinogenic, teratogenic, and mutagenic properties [16]. These by-products may retain their inherent toxic functional groups, thereby inhibiting PPs [15,17].

Currently, details regarding the relationship between NOD-DBPs and PPs are scarce, and a model for NOD-DBP-PP complex interactions has yet to be established. Consequently, the connection between the residual structure of NOD-DBPs and their biological toxicity remains unclear, making it challenging to pinpoint the precise mechanism by which NOD-DBPs potentially inhibit PPs. Given the preceding discussion, this paper aims to explore the potential molecular mechanisms by which prototypical NOD-DBPs inhibit the activity of PP2A. Prototypical NOD-R-DBPs were produced by simulating the chlorine disinfection process, and NOD-R-DBP isomers were separated and identified based on LC, MS, and MS/MS analysis. After chromatographic preparation, the inhibition effect of NOD/NOD-DBPs on PP2A was evaluated by conventional PPs inhibition assay [18]. Based on the similarity between NOD-R and NOD-R-DBPs, the interaction model between NOD-DBPs and the PP2A complex was constructed using a homology modeling strategy. The molecular docking simulation method was adopted to obtain the potential interaction parameters (correlation area, correlation chemical bonding, etc.) between NOD/NOD-DBPs and PP2A. Through correlative analysis of the toxic effects of NOD-DBPs, several key sites and their interactions closely related to the inhibitory effect on PP2A activity were identified. Building on this, we propose that NOD-DBPs suppress PP2A at the molecular level, clarifying its toxic mechanism.

## 2. Results and Discussions

### 2.1. Identification of Prototypical NOD-R-DBPs

**Figure 1:**
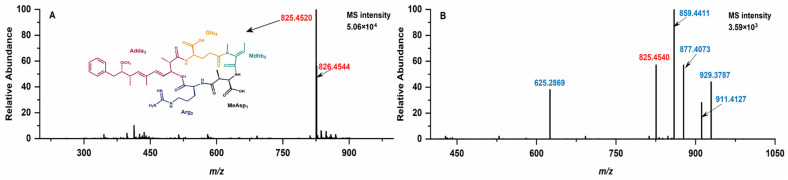
MS spectra of NOD-R (**A**) and its disinfection sample (**B**).

Following chlorination, NOD-R can metamorphose into a variety of prototypical NOD-R-DBPs, each with distinct molecular weights which are identifiable through mass spectrometry analysis (Figure 1). For NOD-R, with a molecular formula of C_41_H_60_N_8_O_10_, the primary mass spectrometry signals are detectable at m/z 825.4520 and 826.4544 (the main isotopic peak) (Figure 1A) [6]. New mass spectral signals of five freshly formed protonated prototypical NOD-R-DBPs were detected at m/z 625.2945, 859.4565, 877.4227, 929.3943, and 911.4281 for the disinfected samples (Figure 1B). With the assistance of *Compass Isotope Pattern* (Version 3.2) software, the chemical formulae of the above NOD-R-DBPs could be determined as C_26_H_40_N_8_O_10_ (-15C, -2OH), C_41_H_62_N_8_O_12_ (+2OH), C_41_H_61_N_8_O_11_Cl (+OH, +Cl), C_41_H_62_N_8_O_12_C_l2_ (+2OH, +2Cl), and C_41_H_63_N_8_O_13_Cl (+3OH, +Cl).

**Figure 2:**
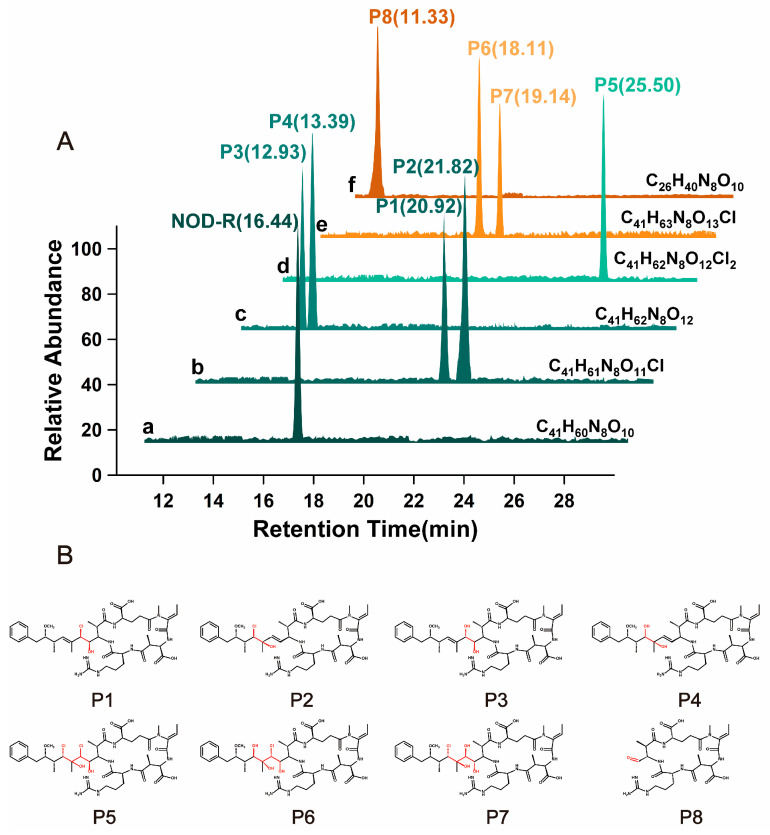
Extract ion chromatograms of native NOD-R and prototypical NOD-R-DBPs from disinfection sample (**A**). The structure of NOD-R-DBPs (P1–P8) (**B**). Conditions: P1–P8 represent oxidized Adda^3^, respectively.

Following solid-phase extraction, the initially identified NOD-R-DBPs in the crude extract underwent purification via preparative chromatographic separation. For NOD-R-DBPs with a specific m/z, multiple isomers with different structures may exist. Therefore, possible isomers of the above NOD-R-DBPs (with the same MS signal) were further identified based on their extracted ion chromatography (EIC) peaks (Figure 2) and characteristic fragment ions. For the protonated NOD-R, an EIC peak was present at around 16.44 min (Figure 2A-a), and its characteristic fragment ions were detected at m/z 135.081, 227.1032, 599.3557, 383.2043, 157.1089, 163.1123, 389.2076, and 691.3779. With the assistance of the above software, the corresponding substructures were identified as [PhCH_2_CH(OCH_3_)]^+^, [Glu^4^-Mdhb^5^ + H]^+^, [Arg^2^-Adda^3^-Glu^4^ + H]^+^, [Mdhb^5^-MeAsp^1^-Arg^2^ + H]^+^, [Arg^2^ + H]^+^, C_11_H_15_O^+^, [C_11_H_15_O-Glu^4^-Mdhb^5^]^+^, and [M + 2H]^+^-[PhCH_2_CH(OCH_3_)]^+^ [19,20].

For prototypical NOD-R-DBPs, eight EIC peaks were eluted (Figure 2A-a–f). By comparing the MS/MS fragment ions associated with the newly formed EIC peaks by NOD-R, Adda^3^ was found to be the major reaction site (Table S1). The generation mechanism of NOD-R-DBPs was clarified through chemical formula analysis: the NOD-R-DBP C_41_H_61_N_8_O_11_Cl had two isomers which were eluted at 20.92 and 21.82 min, and represented P1 and P2, respectively (Figure 2A-b). P1/P2 are likely the addition products formed between 1Cl + 1OH and the conjugated diene Adda^3^. Following Markovnikov’s rule, the chlorine radical would preferentially attach to the carbon atom in the double bond with fewer substituents, while the hydroxyl group would boundto the adjacent carbon [21]. This regioselectivity is characteristic of such electrophilic additions to conjugated systems. Due to steric hindrance, the inner double bond addition product should be less abundant than that of the outer bond. Consequently, P1 and P2 correspond to the products derived from the inner and outer double bonds, respectively. For the NOD-R-DBP C_41_H_62_N_8_O_12_with two EIC peaks at 12.93 and 13.39 min (Figure 2A-c), it can be synthesized by hydroxylating the Adda^3^ diene (2OH addition) or by substituting a chlorine in P1/P2’s diene with a hydroxyl group (1Cl → 1OH). Due to steric hindrance, the inner double bond addition product (P3) is expected to be less abundant than the outer double bond product (P4). Figure 2A-d indicates that NOD-R-DBP C_41_H_62_N_8_O_12_Cl_2_ exhibits only a single EIC peak at 25.50 min. Subsequently, two chlorine atoms and two hydroxyl groups (2Cl + 2OH) can be added to the two double bonds, resulting in the formation of product P5. In addition, P1/P2 can also introduce 1Cl and 1OH onto another C=C double bond of Adda^3^, transforming into P5. Similarly, P3/P4 can also be transformed into P5. In the case of NOD-R-DBP, two distinct EIC peaks appeared at 18.11 and 19.14 min (Figure 2A-e). These peaks, corresponding to the compound NOD-R-DBP C_41_H_63_N_8_O_13_Cl, are believed to align with P6 and P7, respectively. They are likely by-products generated during the formation of P1 to P4. Given the abundance of P1/P2/P3/P4 and the higher abundance of P6, P1 and P4 yield P6 as a secondary product, whereas P2 and P3 produce the less prevalent P7 as a secondary outcome. P5 can be converted into P6/P7 by substitution reaction. For NOD-R-DBP C_26_H_40_N_8_O_10_, which exhibits a single EIC peak at 11.33 min (Figure 2A-f), the decrease in molecular weight means that 15C + 21H has been cleaved from the side chain of Adda^3^ and only 1C + 1H + 1O (P8) is left from the side chain of Adda^3^. P3/P7 can also be converted to P8 by oxidizing the inner double bond to form a C=O bond.

### 2.2. Evaluation of Potential Inhibition Effect

According to the above analysis, evaluating the potential toxicity of NOD-R-DBPs is important for controlling the environmental risk of NOD-R-DBPs. In order to explore their potential toxicity, NOD-R-DBPs were prepared and purified using solid-phase extraction and preparative chromatography. Table S2 presents details on the preparation and purification of NOD-R-DBPs. Because of the high purity (>98.1%) of the prepared NOD-R-DBPs samples, they can be directly used in PPs inhibition tests.

**Figure 3:**
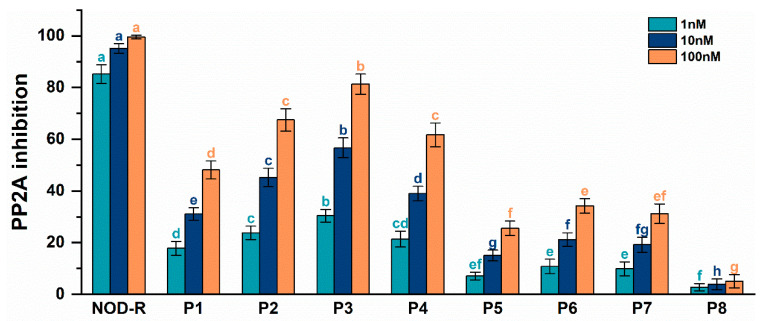
Inhibitory effects of NOD-R and prototypical NOD-R-DBPs on PP2A at different concentrations. The error bar is the standard error of three repeated analyses. Different letters indicate significant differences between groups (*p* < 0.05), obtained using IBM ***SPSS*** Statistics (version 26.0) software [22].

As can be seen in Figure 3, all NOD-R-DBPs at the studied concentrations (1 nM, 10 nM, 100 nM) had potential inhibitory effects on PP2A. Compared with the original toxin, the inhibitory effect of NOD-R-DBPs on PP2A was reduced to varying degrees. The inhibitory effect on PP2A increased with increasing toxin concentration, and a significant dose–effect relationship was observed. There was a significant difference between groups in the inhibitory effects of NOD-R and NOD-R-DBPs on PP2A (*p* < 0.05). At the concentration level of 1 nM, the inhibitory effects of NOD-R and NOD-R-DBPs on PP2A could be categorized into eight classes according to ANOVA analysis: (a) NOD-R; (b) P3; (c) P2; (d) P1; (e) P6, P7; (f) P8; (cd) P4; and (ef) P5. At the concentration level of 10 nM, the inhibition of PP2A by NOD-R and NOD-R-DBPs was categorized into nine classes (a NOD-R; e P1; c P2; b P3; d P4; g P5; f P6; fg P7; h P8) according to ANOVA analysis. At a concentration of 100 nM, the results of ANOVA indicated that NOD-R and NOD-R-DBPs inhibited PP2A in eight distinct groups, with significant differences observed among them (a NOD-R; b P3; c P2, P4; d P1; e P6; ef P7; f P5; g P8). Combining the inhibition data under each concentration condition, the order of inhibition of PP2A by NOD-R and NOD-R-DBPs at the same concentration was obtained: NOD-R > P3 > P2 ≈ P4 > P1 > P6 ≈ P7 ≈ P5 > P8. However, it should be emphasized that NOD-R-DBPs (especially P2, P3, and P4) still have considerable inhibitory effects on PP2A, confirming that NOD-R-DBPs still possess biological toxicity and may, like NOD-R, exhibit hepatotoxicity and carcinogenicity, causing damage to animal livers. Therefore, the secondary environmental risks posed by NOD-R-DBPs warrant further attention.

### 2.3. Simulation of Prototypical NOD-R-DBPs Interaction with PP2A Based on Homology Modeling and Molecular Docking

**Figure 4:**
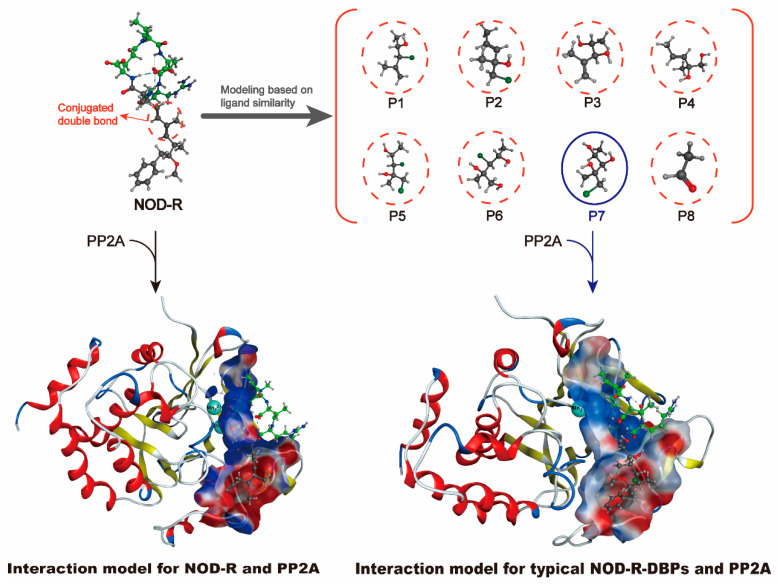
Illustration of interaction model construction for prototypical NOD-R-DBPs–PP2A complexes based on homology modeling strategy.

Since the interaction model of the NOD-R-DBPs–PP2A complex remains unsolved, a deeper understanding of the specific molecular mechanism by which NOD-R-DBPs may inhibit PP2A is limited. Since NOD-R and NOD-R-DBPs have certain structural similarities, the NOD-R–PP2A complex model was considered an ideal template for constructing the NOD-R-DBPs–PP2A complex interaction model. Through homologous modeling strategy [23], the interaction model of NOD-R-DBPs and PP2A complex was constructed (Figure 4). The NOD-R–PP1 model (*PDB code 3E7A*) was downloaded [24,25] and then modified by adding hydrogen atoms and charges. The models for NOD-R and PP2A can be built using homologous modeling, based on the modified NOD-R–PP1 model, where PP2A replaces the original PP1 protein in the modified model. Similarly, prototypical NOD-R-DBPs and PP2A models were constructed: the ligand NOD-R was substituted with the identified NOD-R-DBPs. The “***template dock***” mode was used to dock ligand molecules to the active site of a protein. Developed by ***Molecular Operating Environment software*** (***MOE software***, version 22.02), the “***template dock***” mode is suitable for binding sites where the location is known but specific ligand interaction information is lacking, ensuring the stability and accuracy of the structure [26,27,28]. This process identified 73 potential interaction parameters between NOD-R/NOD-R-DBPs and PP2A, indicating crucial binding sites (Table S3). Among the numerous potential interaction parameters, screening out key interaction parameters related to the toxicity of NOD-R/NOD-R-DBPs is crucial for elucidating the molecular mechanism by which NOD-R/NOD-R-DBPs inhibit PP2A, which is also our next task.

### 2.4. Pearson Correlation Analysis

**Figure 5:**
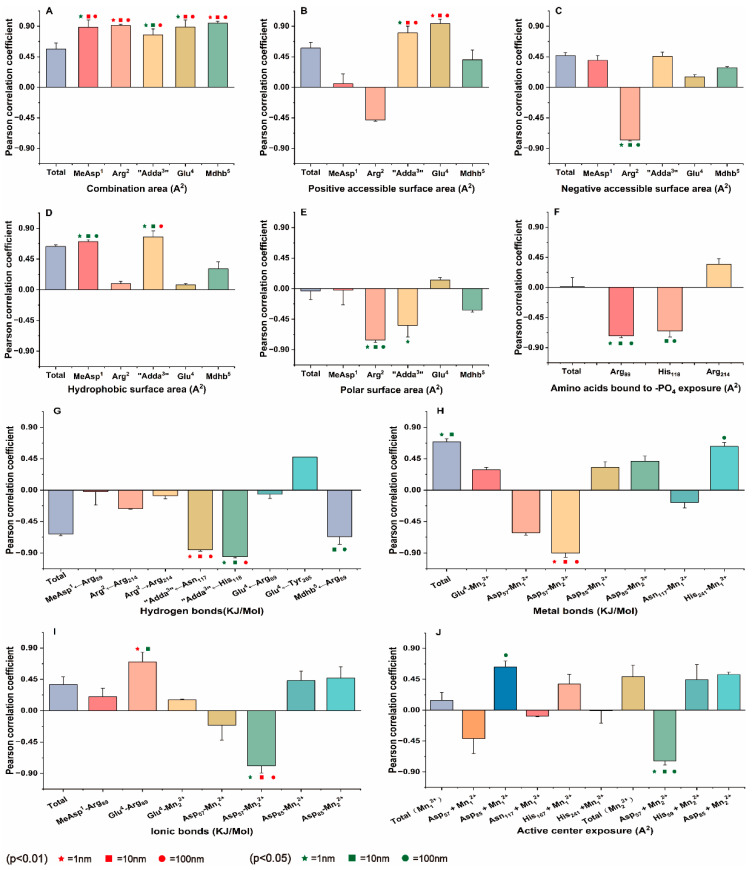
Pearson correlation coefficient between potential interaction parameters and the inhibition data. (**A**) combination area, (**B**) positive accessible surface area, (**C**) negative accessible surface area, (**D**) hydrophobic surface area, (**E**) polar surface area, (**F**) amino acids bound to -PO_4_ exposure, (**G**) hydrogen bonds, (**H**) metal bonds, (**I**) ionic bonds, (**J**) active center exposure. Conditions: 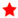, 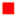, 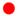 mean that the interaction parameters are extremely and significantly correlated with the inhibition data at the levels of 1, 10, and 100 nM, respectively (*p* < 0.01). 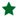, 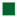, 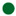 mean that the interaction parameters are significantly correlated with the inhibition data at the levels of 1, 10, and 100 nM, respectively (*p* < 0.05).

How to combine raw analog data with toxicological data to screen for parameters related to toxin toxicity is the next problem we need to solve. Based on the dynamic changes in potential interaction parameters and inhibition data, Pearson correlation analysis was used to evaluate the correlation between potential interaction parameters and inhibition data [29,30,31] (listed in Table S4). To sidestep the elimination of pertinent variables tied to a select-few amino acids, regression analysis was eschewed. The relationship between potential interaction factors and inhibition data was visually depicted in a bar chart. Notably, those interaction factors that exhibited a significant or even an extremely significant correlation with various toxicity levels were denoted with clear, distinguishing symbols (Figure 5). According to Figure 5, potential interaction parameters exhibit varied correlations with inhibitory data across distinct toxin concentration levels [30,32]. At the 1 nM concentration, 46 potential interaction parameters exhibited a positive correlation with the inhibition data, while 26 parameters showed a negative correlation. Six parameters demonstrated an extremely significant correlation (*p* < 0.01) with the inhibition data, and fourteen parameters showed a significant correlation (*p* < 0.05). At the 10 nM concentration, 44 potential interaction parameters correlated positively with the inhibition data, and 28 parameters correlated negatively. Nine parameters were extremely significantly correlated, and twelve were significantly correlated. At the 100 nM concentration, 46 potential interaction parameters correlated positively with the inhibition data, and 26 negatively correlated. Twelve parameters were extremely significantly correlated, and nine were significantly correlated. By comparing the data, it is not difficult to see that there are significant differences in the number of positive and negative correlations, as well as the number of significant and highly significant correlations, across different concentration levels. Additionally, the Pearson correlation coefficients and their significance levels for the same parameter at different concentrations also vary. The interaction parameters demonstrating significant or extremely significant correlations (*p* < 0.05 or *p* < 0.01) with the acquired inhibition data are essential for elucidating the molecular mechanisms underlying the potential inhibitory effects of NOD-R-DBPs on PP2A. Therefore, the next research question is how to organize and further screen for key parameters that play a significant role.

**Figure 6:**
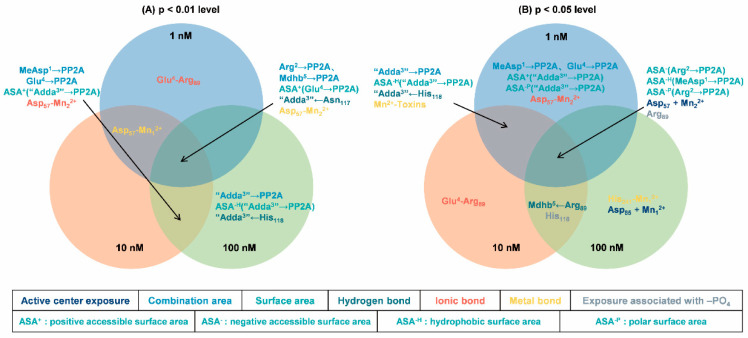
Venn diagrams of the significant interaction parameters at the *p* < 0.01 level (**A**) and *p* < 0.05 level (**B**).

At multiple concentrations, data pairs with strong correlations with toxicity data are more important for analyzing mechanisms. To identify key interaction parameters amidst the diverse range of correlations, Venn diagrams were employed (Figure 6). Venn diagrams can help us understand the correlation between parameters more intuitively, making it easier to screen and confirm parameters with stronger correlations [33,34]. At a significance level of *p* < 0.01, five interaction parameters had extremely significant correlations with the inhibition data at three toxin concentrations (for example, hydrogen bond for “Adda^3^” ← Asn_117_). Five parameters have shown extremely significant correlation with inhibition data at two toxin concentrations (such as the positive accessible surface area for “Adda^3^” → PP2A). Four parameters had extremely significant correlations with inhibition data at one toxin concentration (for example, ionic bond Glu^4^-Arg_89_). At a significance level of *p* < 0.05, five interaction parameters have shown extremely significant correlation with inhibition data across three toxin concentrations (for example, the negative accessible surface area for Arg^2^ → PP2A). Six parameters had extremely significant correlations with inhibition data at two toxin concentrations (such as hydrogen bond for “Adda^3^” ← His_118_ and the exposure area of amino acid His_118_ bound to -PO_4_). Eight parameters have shown extremely significant correlation with inhibition data at one toxin concentration (such as the combination area for MeAsp^1^ → PP2A, ionic bond Glu^4^-Arg_89_,and the catalytic center exposure area for Asp_85_ + Mn_1_^2+^). Evidently, the aforementioned interaction parameters, particularly those correlated with inhibition data, are instrumental in elucidating the molecular mechanism underlying the potential inhibitory effects of NOD-R-DBPs on PP2A.

**Figure 7:**
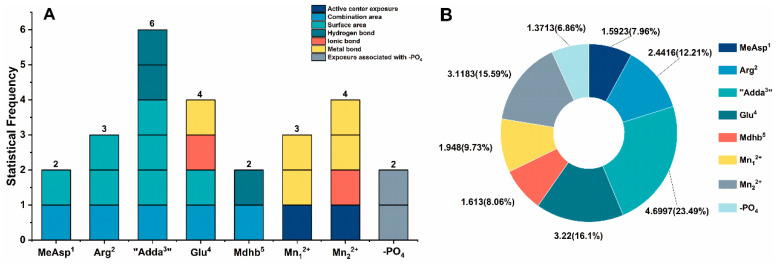
Histogram of the frequency of crucial interaction sites (**A**) and pie chart of the total |$\bar{R}$| values of crucial interaction sites (**B**). Conditions: $\bar{R}$ is the average Pearson correlation under three toxin concentrations.

The toxicity of NOD-R/NOD-R-BDPs is closely related to their interaction sites. Based on the structural units of NOD-R/NOD-R-DBPs, the catalytic center Mn^2+^, and the introduced -PO_4_, statistical analysis was performed on the key interaction parameters to identify critical interaction sites (Figure 7). Among them, the metal bonds for Mn^2+^-Toxins should be attributed to Glu^4^, Mn_1_^2+^, and Mn_2_^2+^. Statistical frequency analysis (Figure 7A) showed that six of the above key interactions were associated with “Adda^3^”, four parameters were associated with Glu^4^/Mn_2_^2+^, three parameters were associated with Arg^2^/Mn_1_^2+^, and two parameters were associated with MeAsp^1^/-PO_4_/Mdhb^5^. The total |$\bar{R}$| values of the critical interaction sites were statistically analyzed. It was found that “Adda^3^”, Glu^4^, Mn_2_^2+^, Arg^2^, Mn_1_^2+^, Mdhb^5^, MeAsp^1^, and -PO_4_ were all involved in the combination of NOD-R/NOD-R-DBPs to PP2A, and their contributions tended to decrease. Specifically, “Adda^3^” has a significant impact on the binding of NOD-R/NOD-R-DBPs with PP2A, Glu^4^/Mn_2_^2+^/Arg^2^/Mn_1_^2+^ have a greater impact on the binding of NOD-R/NOD-R-DBPs with PP2A, and MeAsp^1^/-PO_4_/Mdhb^5^ have a certain impact on the binding of NOD-R/NOD-R-DBPs with PP2A (Figure 7B). Among the structural units of NOD-R/NOD-R-DBPs, “Adda^3^” was involved in significantly more key interaction parameters than other interaction sites. Therefore, it is hypothesized that the alteration of “Adda^3^” during chlorination is the key to the significant reduction in NOD-R toxicity. In addition, the present study found that Glu^4^ also had a significant influence on the toxicity of NOD-R/NOD-R-DBPs.

Adda^3^ is a special amino acid which plays a decisive role in the toxicity of NODs. Previous studies have found that Adda^3^ has an important effect on the toxicity of NOD-R/NOD-R-DBPs. The conclusion of this study is basically consistent with previous research results. In this study, it was found that in the structural unit of NOD-R/NOD-R-DBPs, “Adda^3^” involved more key interaction parameters than other interaction sites.

### 2.5. Molecular Mechanism Analysis

**Figure 8:**
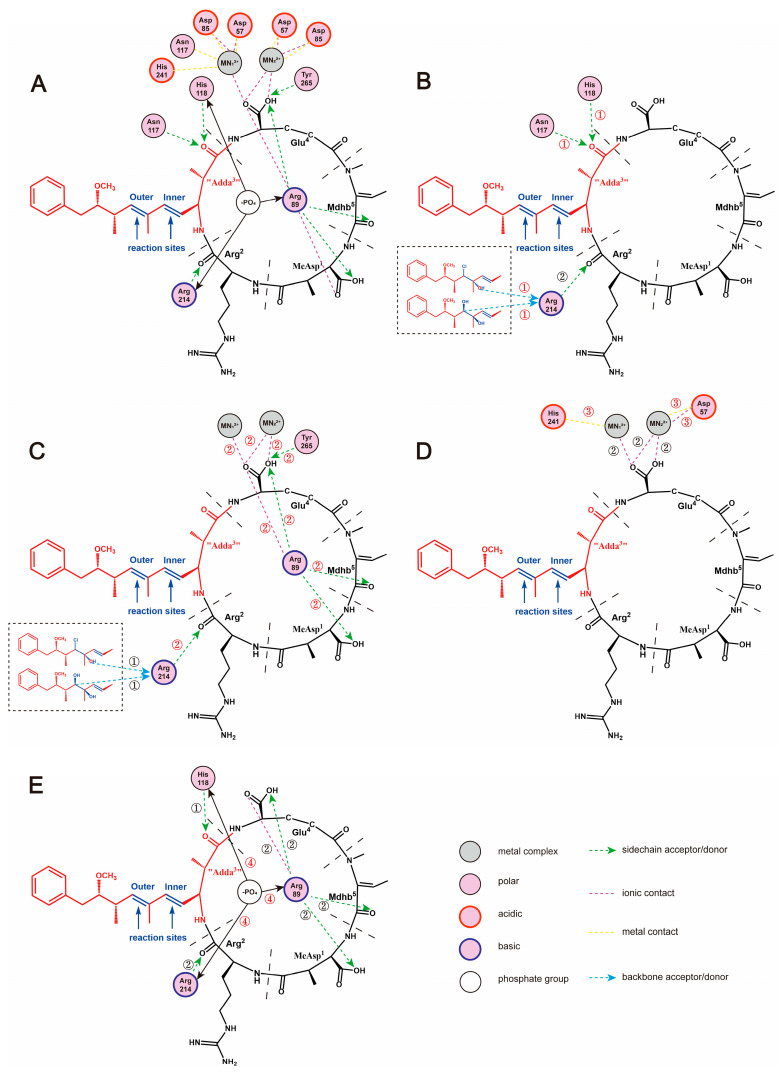
The 2D ligand-receptor interaction diagram for the combination of the toxins to PP2A. The interaction between NOD-R and PP2A (**A**). The interaction between toxins and PP2A that is directly affected by the damaged “Adda^3^” (**B-➀**). The interaction between toxins and PP2A that is indirectly affected by the damaged “Adda^3^” (**C-➁**). Influenced interactions related to Mn^2+^ ions (**D-➂**). Effect on the exposure of amino acids bound to phosphate group (**E-➃**).

A 2D ligand-receptor interaction diagram offered better ways to illustrate the key interaction parameters. The key interactions between NOD-R/NOD-R-DBPs and PP2A include hydrogen bonds “Adda^3^” ← Asn_117_, “Adda^3^” ← His_118_, Mdhb^5^ ← Arg_89_; ionic bonds Glu^4^-Arg_89_, Asp_57_-Mn_2_^2+^; and metal bonds Asp_57_-Mn_2_^2+^, His_241_-Mn_1_^2+^ (Figure 8). Obviously, NOD-R-DBPs retain the potential to inhibit PP2A because they preserve all or some of the crucial interaction sites, referred to as key interactions. As the chlorination reaction proceeded, the structure of Adda^3^ was gradually disrupted, leading to a general decrease in the inhibitory effect of NOD-R-DBPs on PP2A. Apparently, structural differences altered the above key interactions, thereby reducing the potential inhibitory effects of NOD-R-DBPs on PP2A.

Combining the above, the added polar groups·Cl and·OH initially weaken the hydrophobic interactions and positive electrostatic interactions between “Adda^3^” and PP2A, consequently weakening the binding of “Adda^3^” and PP2A. The combination area, positive accessible surface area, and hydrophobic surface area related to “Adda^3^” are positively correlated with toxicity. The disruption of “Adda^3^” directly weakened the hydrogen bonds “Adda^3^” ← Asn_117_ and “Adda^3^” ← His_118_ (Figure 8B). Correspondingly, the combination area of Adda^3^ → PP2A also showed a decreasing trend. Subsequently, the disruption of “Adda^3^” also interfered with key interactions between other structural units of NOD-R-DBPs and PP2A (Figure 8C), including the weakened hydrogen bond Mdhb^5^ → Arg_89_ and the strengthened ionic bond Glu^4^-Arg_89_, which resulted in a weakening of the binding of NOD-R-DBPs to PP2A. The corresponding is the combination area of Mdhb^5^ → PP2A shows a consequent decrease. However, it is possible that the combination area of Glu^4^ to PP2A did not increase, likely due to the competitive effect of the catalytic center Mn^2+^. Subsequently, the side chain of Glu^4^ could interact with the catalytic center Mn^2+^ and form a new metal bond Glu^4^-Mn_1_^2+^ (Figure 8C). The observed alterations in these critical interactions further diminished the ionic bond Asp_57_-Mn_2_^2+^ in the conserved region of PP2A and Mn^2+^ (Figure 8D).

Previous studies indicated the catalytic core of PP2A contains nine strictly conserved amino acids [35], six of which are coordinated to Mn^2+^ (His_59_, His_167_, His_241_, Asp_57_, Asp_85_, Asn_117_), and three of which are bound to -PO_4_ (Arg_89_, His_118_, Arg_214_) (Figure 8E). For the interactions involved in catalytic substrates, the weakening of ionic bond Asp_57_-Mn_2_^2+^ promoted the exposure of Mn_2_^2+^ coordinated to Asp_57_. In addition, changes in the interactions between the three conserved amino acids and NOD-R-DBPs interfered with the combination of the conserved amino acids to -PO_4_. Due to the weakening of hydrogen bonds “Adda^3^” ← His_118_, Mdhb^5^ ← Arg_89_, the combination of His_118_ and Arg_89_ to -PO_4_ was significantly enhanced, whereas the combination of Arg_214_ to -PO^4^ was not significantly affected. The recovery of PP2A catalytic activity was caused by the increase in both Mn^2+^ exposure and the combination of conserved amino acids to -PO_4_.

## 3. Conclusions

In this study, we investigated the potentially toxic effect of prototypical NOD-R-DBPs target on PP2A. After disinfection treatment, NOD-R underwent oxidation, generating five NOD-R-DBPs with slightly different molecular weights. Multiple NOD-R-DBPs were isolated and identified based on MS, LC/MS, and MS/MS cascade techniques, indicating that the conjugated diene in Adda^3^ was the main reactive site. PP2A inhibition assay showed that NOD-R/NOD-R-DBPs inhibited PP2A in the following order: NOD-R > P3 > P2 ≈ P4 > P1 > P6 ≈ P7 ≈ P5 > P8. Using homology modeling, an interaction model for the prototypical NOD-R-DBPs–PP2A complex was built, based on the NOD-R–PP2A complex. Based on molecular simulation experiments, the interaction parameters of NOD-R/NOD-R-DBPs with PP2A were obtained. Pearson correlation analysis was conducted on the inhibition test data and molecular simulation parameters to identify key interaction parameters related to the toxicity of NOD-R-DBPs, and the molecular mechanism of NOD-R-DBPs’ potential inhibitory effects on PP2A was elucidated: the disruption of “Adda^3^” directly led to the weakening of the hydrogen bonds “Adda^3^” ← Asn_117_ and “Adda^3^” ← His_118_. Subsequently, the disruption of “Adda^3^” altered the key interactions between NOD-R-DBPs and PP2A, including a weakened hydrogen bond Mdhb^5^ → Arg_89_, a strengthened ionic bond Glu^4^-Arg_89_, and a strengthened metal bond Glu^4^-Mn_2_^2+^. The above changes in the interactions further modified the interaction between the conserved amino acids and the catalytic center Mn^2+^ ions, including the weakened ionic bond Asp_57_-Mn_2_^2+^. Meanwhile, the weakening of hydrogen bonds “Adda^3^” ← His_118_ and Mdhb^5^ ← Arg_89_ increased the exposure of amino acids His_118_ and Arg_89_, thus promoting the combination of -PO_4_ to His_118_ and Arg_89_. These key interactions determine the exposure of the catalytic center and the recovery of the catalytic activity of PP2A. Prototypical NOD-R-DBPs retained the aforementioned key functional sites, suggesting that they still possess the potential to inhibit PP2A. As the chlorination reaction proceeded, Adda^3^ was disrupted to varying extents, and the above key interactions changed accordingly, leading to differences in the inhibitory effects of different NOD-R-DBPs on PP2A.

## 4. Materials and Methods

### 4.1. Materials

NOD-R was purchased from Sigma (Saint-Quentin Fallavier, France). PP2A was obtained from New England Biolabs Inc. Na_2_S_2_O_3_, MgCl_2_, MnCl_2_, HCl, Ca(ClO)_2_, high-purity CO_2_, p-Nitrophenyldisodium orthophorphate (p-NPP), tris(hydroxymethyl) aminomethane (Tris), bovine serum albumin (BSA), dithiothreitol (DTT), neoprene rubber, sodium nitrobenzene disodium, and ascorbic acid were purchased from Sinopharm (Shanghai, China). HCOOH, CH_3_OH, CF_3_COOH, and HPLC acetonitrile were purchased from Merck (Darmstadt, Germany).

### 4.2. Chlorination Treatment of NOD-R

The chlorination and disinfection of NOD-R was performed using hypochlorous acid (HClO). HClO was generated through the reaction of calcium hypochlorite with carbon dioxide [36]. At room temperature, 250 mL NOD-R (100 μg/L) and 250 mL HClO (about 4 mg/L) were mixed in a brown reagent bottle and reacted in the dark. The reaction time was controlled by adding an amount of termination reagent (10 mg/L ascorbic acid stock solution). For the detection of NOD-R-DBPs, a 5 mL sample from the reaction system was usually taken and mixed thoroughly with 1 mL of ascorbic acid solution. In contrast, during the preparation of NOD-R-DBPs, 50 mL of ascorbic acid solution was added to the reagent bottle to effectively quench the reaction. Each test group included a control group that utilized ultrapure water in place of HClO and termination reagent.

### 4.3. Purification and Preparation of Prototypical NOD-R-DBPs

#### 4.3.1. MS Analysis

The chlorinated samples were analyzed by a maXis UHR-TOF mass spectrometer. The chlorinated samples were mixed with an equal volume of acetonitrile (containing 0.1% trifluoroacetic acid) and then injected into the mass spectrometer at a rate of 3 μL/min through an autosampler. The MS parameters were set as follows: positive ion spray ionization pattern, capillary voltage of 3.8 kV, sampling cone/orifice voltage of 0.45 kV, nebulizing gas (N_2_) 0.5 bar, dry gas (N_2_) heater 200 °C, dry gas (N_2_) flow rate 4 L/min, and the scanning range was 150.00001–1049.99999. The data acquisition was performed by Compass 1.3 software, which led to the preliminary identification of NOD-R-DBPs.

#### 4.3.2. Purification of Prototypical NOD-R-DBPs

The purification of NOD-R-DBPs was carried out by borrowing the traditional method of concentration and enrichment of MCs [37,38]. The C_18_ solid-phase extraction column, washed with 10 mL of pure acetonitrile and 10 mL of ultrapure water, was used for sample pretreatment. As elution solvents, 5 mL of 20% acetonitrile and 5 mL of 80% acetonitrile were used, respectively, to sequentially remove impurities and enrich NOD-R-DBPs targets. The collected crude extracts of NOD-R-DBPs were evaporated to dryness under N_2_ and reconstituted with 200 µL of 20% acetonitrile solution. Subsequently, the extracts were separated by chromatography in a HPLC-MS coupled system equipped with a Great Eur-Asia C_18_ column (9.4 × 250 mm, 5 um, 120 Å) [11]. Mobile phase A was configured as an ultrapure water solution containing 0.1% trifluoroacetic acid, while mobile phase B utilized an acetonitrile solution containing 0.1% trifluoroacetic acid. For the separation of NOD-R-DBPs, a gradient elution was employed, starting with 20% mobile phase B for 5 min, followed by a linear increase in mobile phase B proportion from 20% to 80% over 20 min. Subsequently, an isocratic elution was performed for 5 min, after which the concentration of mobile phase B was rapidly decreased to 20% within 0.1 min, and the column was then equilibrated for 5 min. During the experiment, the column temperature was maintained at 30 °C, and the flow rate was controlled at 5 mL/min.

#### 4.3.3. Preparation of Prototypical NOD-R-DBPs

Using an autosampler, a portion of the chromatographic mobile phase was directed into the high-resolution mass spectrometer for analysis. The mass spectrometry experimental parameters were largely consistent with those detailed in Section 4.3.1, with the exception that the analysis mode was switched from “full scan” to “selective ion scan”. Confirmatory analysis of NOD-R-DBPs was conducted utilizing specific retention times and chromatographic peaks. The chromatographically separated NOD-R-DBPs were collected in centrifuge tubes at their specific retention times and subsequently dried by N_2_ rotary evaporation, and the change in mass of the centrifuge tubes was recorded using a PL2002 electronic balance (Mettler Toledo, Shanghai, China). Finally, NOD-R-DBPs were dissolved in 200 μL of acetonitrile [11]. The structure of NOD-R-DBPs was further characterized by comparing the characteristic fragment ions of NOD-R-DBPs with those of NOD-R on the basis of MS/MS analysis. The parameters of MS/MS were the same as those of Section 4.3.1, except that the collision energy of the secondary process was set to 55 eV.

### 4.4. PP2A Inhibition Assay

We used standard protein phosphatase inhibition experiments to detect the inhibitory effect of prototypical NOD-R-DBPs on PP2A activity [39]. First, a solution containing a 50 mM Tris-HCl buffer system (pH 7.4) was used, to which 1.0 mM MnCl_2_ and 2.0 mM dithiothreitol were added as the reaction medium. Simultaneously, 1.0 g/L BSA was added to dilute the PP2A to a working concentration of 5 U/mL. Subsequently, 10 μL of enzyme solution and 100 μL of the sample to be tested were added into separate wells of a 96-well polystyrene microplate and gently shaken to mix and facilitate the reaction. Incubation was performed for 15 min at a steady 25.0 °C temperature. Subsequently, 90 μL p-NPP solution was added. Following a 60 min reaction, the optical density of the enzyme substrate at a wavelength of ODS_405_ was detected using a microplate reader. The relative PP2A enzyme activity was calculated as a percentage using the following formula: I_PP2A_ (%) = (A_toxins_ − A_blank_)/(A_control_ − A_blank_) × 100%. Distilled water was used in place of the toxin in the control group. In the blank group, distilled water replaced both the NOD-R or NOD-R-DBPs and the PP2A enzyme solution.

### 4.5. Molecular Simulation

Molecular docking simulation was performed using ***MOE software*** [11]. The NOD-R–PP1 complex model (*PDB code 3E7A*) was obtained from Protein Data Bank. Upon importing the complex model into ***MOE software*** for further analysis, missing loop regions were then reconstructed using the “building missing loops” feature, and the charge distributions for both NOD-R and PP2A were systematically optimized [40]. Based on the modified model of NOD-R–PP1, the model of NOD-R and PP2A could be constructed by homologous modeling: the original protein phosphatase catalytic subunit 1 in the modified model was replaced by 2A. Following the same approach, the original ligand NOD-R was substituted with various NOD-R-DBPs to build models of prototypical NOD-R-DBPs and PP2A [40,41]. The NOD-R-DBPs–PP2A models were optimized for energy efficiency, followed by molecular docking simulations of NOD-R/NOD-R-DBPs with PP2A. To maintain consistency between NOD-R-DBPs and their original toxins, the “***template dock***” approach was employed. In this method, the positioning and optimization of NOD-R-DBPs mirrored that of their original counterparts exactly [41]. Identified potential interaction parameters for the toxin-PP2A complex.

## Data Availability

The original contributions presented in this study are included in the article and Supplementary Material. Further inquiries can be directed to the corresponding author.

## Supplementary Materials

The following supporting information can be downloaded at https://www.mdpi.com/article/10.3390/toxins17100484/s1, Table S1: MS/MS identification of NOD-R and NOD-R-DBPs; Table S2: Preparation and purification information for typical NOD-R-DBPs; Table S3: The candidate interaction parameters between NOD-R/NOD-R-DBPs and PP2A; Table S4: Correlation between candidate interaction parameters and inhibition data.
