# Supplementary material for: Evaluating the Potential Inhibition of PP2A by Nodularin-R Disinfection By-Products: Effect and Mechanism
Source: Toxins (Basel). 2025 Sep 26;17(10):484. doi: 10.3390/toxins17100484 (PMC12567575; doi:10.3390/toxins17100484)
Supplement: Supplementary file 1 [file toxins-17-00484-s001.zip › toxins-3788921-supplementary.pdf]

Table S1. MS/MS identification of NOD-R and NOD-R-DBPs

Basic fragment ions	NOD-R	$C_{41}H_{60}N_8O_{12}Cl_2$	$C_{26}H_{40}N_8O_{10}$	$C_{41}H_{59}N_8O_{11}Cl$		$C_{41}H_{60}N_8O_{12}$		$C_{41}H_{61}N_8O_{13}Cl$	
		P5	P8	P1	P2	P3	P4	P6	P7
$[M+H]^+$	825.4540	929.3787↑ [a]	625.2869↓	877.4073↑	877.4073↑	859.4411↑	859.4411↑	911.4127↑	911.4127↑
$[PhCH_2CH(OCH_3)]^+$	135.081	135.081 √ [b]	--- [c]	135.081 √	135.081 √	135.081 √	135.081 √	135.081 √	135.081 √
$[Glu\text{-}Mdhb+H]^+$	227.1032	227.1032 √	227.1032 √	227.1032 √	227.1032 √	227.1032 √	227.1032 √	227.1032 √	227.1032 √
$[Arg\text{-}Adda\text{-}Glu+H]^+$	599.3557	701.2833↑	398.1914↓	649.3117↑	649.3117↑	631.3455↑	631.3455↑	683.3172↑	683.3172↑
$[Mdhb\text{-}MeAsp\text{-}Arg+H]^+$	383.2043	383.2043 √	383.2043 √	383.2043 √	383.2043 √	383.2043 √	383.2043 √	383.2043 √	383.2043 √
$[Arg+H]^+$	157.1089	157.1089 √	157.1089 √	157.1089 √	157.1089 √	157.1089 √	157.1089 √	157.1089 √	157.1089 √
$C_{11}H_{15}O^+$	163.1123	265.0399↑	97.029↓	213.0683↑	213.0683↑	195.1021↑	195.1021↑	247.0738↑	247.0738↑
$[C_{11}H_{15}O\text{-}Glu\text{-}Mdhb]^+$	389.2076	491.1352↑	323.1243↓	439.1636↑	439.1636↑	421.1974↑	421.1974↑	473.1691↑	473.1691↑
$[M+2H]^+ - [PhCH_2CH(OCH_3)]^+$	691.3779	793.3055↑	625.2946↓	741.3339↑	741.3339↑	723.3677↑	723.3677↑	775.3394↑	775.3394↑
Target residues	---	Adda							

[a]: ↑ and ↓ mean mass changes were related to these fragment ions;

[b]: √ means ions with the stable m/z were detected by mass spectrograph;

[c]: --- no related parameter was detected.

Table S2. Preparation and purification information for typical NOD-R-DBPs

NOD-R-DBPs	Disinfection times	LC collection times	Concentration [a]	Purity [b]
P1	20-60 mins	20.92± 0.25 min	≈52.4 μmol/L	98.5%
P2	20-70 mins	21.82± 0.25 min	≈64.3 μmol/L	99.3%
P3	30-90 mins	12.93± 0.20 min	≈87.1 μmol/L	99.1%
P4	30-90 mins	13.39± 0.20 min	≈112.9 μmol/L	99.4%
P5	20-50 mins	25.50 ± 0.25min	≈126.4 μmol/L	99.3%
P6	20-60 mins	18.11± 0.25 min	≈29.2 μmol/L	98.1%
P7	20-60 mins	19.14± 0.25 min	≈35.8 μmol/L	99.2%
P8	20-50 mins	11.33± 0.25 min	≈78.2 μmol/L	99.6%

[a:] The concentrations of typical NOD-R-DBPs were directly calculated according to their MS signals compared with that of NOD-R. NOD-R and NOD-R-DBPs were assumed to have approximate protonated efficiencies;

[b:] the purity of typical NOD-R-DBPs was directly calculated according to their MS intensities and defined as $I_{Pi}/(I_{NOD\text{-}R}+\sum I_{Pi}) \times 100\%$.

Table S3. The candidate interaction parameters between NOD-R/NOD-R-DBPs and PP2A

Molecular simulation parameters		NOD-R	P1	P2	P3	P4	P5	P6	P7	P8
Combination energy (KJ/Mol)		-4915.52 [a]	-4822.46	-4895.84	-4890.14	-4835.33	-4813.5	-4826.42	-4816.75	-4892.68
Combination area (Å^2)	Total	588.4475	553.1586	554.2195	541.5883	550.3582	529.921	557.8593	576.2477	380.8511
	MeAsp^1→PP2A	59.2964	55.6781	57.5953	57.4795	58.1233	53.2987	54.4089	54.4610	51.1182
	Arg^2→PP2A	103.1024	94.7034	96.75123	94.0324	97.0514	86.9907	91.5240	91.8266	90.8817
	Adda^3→PP2A	395.1255	334.0780	343.8309	340.7950	347.1599	301.7806	318.5047	319.3655	178.6493
	Glu^4→PP2A	160.6725	145.1868	156.6541	152.7451	158.5129	140.6296	144.0734	145.0119	135.9206
	Mdhb^5→PP2A	108.1885	105.2056	106.0544	105.3031	106.5597	104.7754	105.0391	105.0567	103.2819
Positive accessible surface area (Å^2)	Total	340.9193	316.9556	307.0149	327.9247	322.6152	321.9668	315.3567	322.8139	227.3576
	MeAsp^1→PP2A	26.2473	28.8437	26.4479	26.5319	29.6244	26.7712	27.0542	26.9472	26.4673
	Arg^2→PP2A	57.9077	70.9716	62.8330	62.1580	59.8127	56.1200	73.8145	75.4784	67.1679
	Adda^3→PP2A	258.3787	203.6221	228.8810	218.5880	233.6568	190.2721	202.3048	206.3592	110.4162
	Glu^4→PP2A	97.6374	91.6244	92.0654	92.0080	93.4758	91.1613	91.5684	91.1197	90.2112
	Mdhb^5→PP2A	66.6319	64.3275	64.4174	64.3181	64.8025	63.2930	64.8656	62.9562	66.3655
Negative accessible surface area (Å^2)	Total	241.6516	225.0217	237.7642	223.6636	225.5665	245.5583	232.5024	243.4337	153.4949
	MeAsp^1→PP2A	27.9685	26.8862	28.8112	28.3995	28.4986	28.5193	28.3465	27.5056	26.8096
	Arg^2→PP2A	27.4998	29.6846	31.9183	29.0825	28.0305	31.3907	31.7109	29.3474	33.0590
	Adda^3→PP2A	135.9615	130.4481	130.2162	132.3235	130.5030	148.9335	125.8725	141.4881	47.6759
	Glu^4→PP2A	68.2009	65.0544	68.5360	67.7589	60.6308	62.2973	67.7410	66.8922	65.1251
	Mdhb^5→PP2A	42.5091	43.3531	41.2062	40.3477	43.1301	39.9854	43.5598	43.5906	39.9180
Hydrophobic surface area (Å^2)	Total	334.3635	257.2744	272.3515	282.7859	267.3806	298.8830	275.6593	279.3454	164.1702
	MeAsp^1→PP2A	5.2554	5.4593	4.2683	3.6535	4.4639	3.0840	3.0150	3.5786	3.2195

	Arg^{2}→PP2A	45.3947	42.9120	43.3216	43.1547	44.9007	41.7822	50.7538	43.6441	43.3773
	Adda^{3}→PP2A	239.9205	182.8331	190.458	188.3008	209.9625	165.1245	176.6148	180.1721	59.2102
	Glu^{4}→PP2A	44.6741	43.9343	45.7854	45.6692	44.9850	45.0044	42.4686	43.0169	46.3713
	Mdhb^{5}→PP2A	53.5225	50.1513	51.6581	51.1400	52.8316	53.4566	50.6837	50.0652	52.9611
Polar surface area (Å^{2})	Total	248.2064	263.4367	260.8018	264.9430	265.5729	278.6420	272.1998	286.8950	216.6807
	MeAsp^{1}→PP2A	50.2169	52.5404	52.1270	51.7461	53.6591	52.2145	52.3936	50.8824	50.0559
	Arg^{2}→PP2A	43.8114	54.7835	53.2297	48.2778	43.9535	49.6443	54.7715	56.1745	55.5041
	Adda^{3}→PP2A	87.5104	152.9437	144.7194	147.6823	142.4371	160.1274	160.3815	155.9866	109.7472
	Glu^{4}→PP2A	113.6406	110.3763	114.5043	113.5106	113.1216	108.4542	121.8402	112.7950	110.2093
	Mdhb^{5}→PP2A	54.7061	55.5292	60.7655	52.3177	54.1008	51.4211	62.7418	62.6824	59.3214
Hydrogen bonds (KJ/Mol)	Total	-28.5	-20.16	-26.66	-25.74	-25.2	-18.6	-22.4	-22.8	-25.34
	MeAsp^{1}←Arg$_{89}$	-1.08	-2.4	-1.4	-1.96	-2.92	-1.38	-1.6	-1.84	-1.28
	Glu^{4}←Arg$_{89}$	-3.325	-4	-3.26	-3.43	-2.4	-2.1	-2.78	-2.43	-3.06
	Mdhb^{5}←Arg$_{89}$	---[b]	---	-0.9	-0.5	-1.767	---	---	-1.5	---
	Arg^{2}←Arg$_{214}$	---	-0.6	-0.6	---	---	---	---	---	---
	Arg^{2}→Arg$_{214}$	-3.4	-2.8	-2.85	-2.84	-2.7	-2.5	-2.725	-2.8	-2.2
	Adda^{3}←Asn$_{117}$	-1.5	-1	---	---	---	---	-0.8	-0.6	-0.5
	Adda^{3}←His$_{118}$	-17.2	-14.56	-17.26	-17.68	-14.525	-15.48	-17.46	-17.54	-16.36
	Glu^{4}←Tyr$_{265}$	-0.6	-0.675	---	---	---	---	-0.62	-0.98	---
	Arg^{2}→Pro$_{213}$	-2.14	-1.63	-2.16	-2.04	-2.1	-1.96	-1.86	-1.4	-1.24
Ionic bonds (KJ/Mol)	Total	-79.74	-77.86	-84.18	-85.38	-66.18	-83.16	-84.62	-78.14	-85.72
	MeAsp^{1}-Arg$_{89}$	-4.3	-8	-4.88	-5.6	-8.72	-5.58	-6.1	-5.52	-6.32
	Glu^{4}-MN$_{2}^{2+}$	-9.56	-11.4	-11.42	-11.5	-10.8	-10.68	-11.3	-11.16	-11.34

	Glu^{4}-Arg_{89}	-8.68	-8.3	-8.98	-9.38	-8.4	-8.3	-8.62	-9.34	-9.16
	Asp_{57}-MN_1^{2+}	-10.1	-10.59	-10.63	-10.61	-10.7	-10.41	-10.49	-10.55	-9.4
	Asp_{85}-MN_1^{2+}	-15.6	-15.5	-15.5	-15.5	-15.5	-15.2	-15.4	-15.4	-15.1
	Asp_{57}-MN_2^{2+}	-19.3	-20	-20.3	-20.3	-20.85	-20.1	-20.1	-19.8	-20.7
	Asp_{85}-MN_2^{2+}	-12.2	-12.3	-12.3	-12.4	-12.3	-12.3	-12.3	-12.3	-12.3
Metal bonds (KJ/Mol)	Total	-30.1	-33.2	-31.9	-32	-30.5	-32.5	-31.54	-31.8	-32
	MN_2^{2+}-Glu^{4}	-2.7	-3.175	-2.9	-3.12	-2.66	-2.6	-2.74	-3.06	-3.02
	Asp_{57}-MN_1^{2+}	-5.06	-5	-5	-5	-4.9	-4.9	-5	-4.9	-4.9
	Asp_{85}-MN_1^{2+}	-5.6	-5.5	-5.5	-5.5	-5.5	-5.4	-5.4	-5.4	-5.3
	Asn_{117}-MN_1^{2+}	-6	-6.15	-6.1	-6.1	-6.2	-5.95	-6.17	-6.17	-6.2
	His_{241}-MN_1^{2+}	-4.6	-4.6	-4.6	-4.6	-4.7	-4.7	-4.6	-4.6	-4.8
	Asp_{57}-MN_2^{2+}	-2.9	-2.9	-2.9	-2.8	-3	-3	-2.8	-3.2	-2.6
	Asp_{85}-MN_2^{2+}	-4.8	-4.9	-4.8	-4.8	-5	-5	-5	-5.2	-5.1
Exposure areas associated with -PO_4 (Å^2)	Arg_{89}+His_{118}+Arg_{214}	1027.4954	1025.4478	1021.7849	1022.9242	1015.5502	1021.8691	1030.4685	1026.0212	1021.1765
	Arg_{89}	374.4583	377.7976	376.4290	376.9300	378.8776	378.4295	378.7448	376.4083	379.9952
	His_{118}	320.5391	321.7867	320.5375	320.4488	320.3586	322.276	321.7313	321.4715	321.2241
	Arg_{214}	373.773	373.196	374.7982	374.0975	373.3239	374.1201	374.2791	369.8413	371.4368
Active center exposure (Å^2)	Mn_1^{2+}+Asp_{57}+Asp_{85}+Asn_{117}+His_{167}+His_{241}	800.3077	796.264	795.223	798.9213	800.5188	798.2334	797.7987	798.7466	800.9664
	Mn_1^{2+}+Asp_{57}	319.5022	318.4189	318.2344	318.8833	318.2017	320.339	319.3366	319.1235	320.7408
	Mn_1^{2+}+Asp_{85}	309.6601	309.9315	308.8113	309.1113	309.0588	308.9016	308.2703	308.5852	306.6993
	Mn_1^{2+}+Asn_{117}	327.8768	327.6382	327.1212	328.2877	329.7357	327.8007	327.4362	327.9546	329.6188
	Mn_1^{2+}+His_{167}	386.2784	385.9449	385.8559	385.8201	385.3338	385.5953	386.0551	385.5401	385.7596
	Mn_1^{2+}+His_{241}	342.0237	339.5203	338.2462	339.7744	340.1754	339.5718	339.6631	340.9644	341.5836
	Mn_2^{2+}+Asp_{57}+His_{59}+Asp_{85}	567.0465	566.6201	567.4017	568.4137	567.611	567.0748	566.2724	566.2609	565.6704

	Mn_2^{2+}+Asp_{57}	309.5977	309.8842	310.9455	312.2344	310.0031	311.7861	311.7363	311.3728	313.4212
	Mn_2^{2+}+His_{59}	347.0356	346.9065	348.7223	348.8097	349.4545	346.599	346.7025	346.3594	346.1476
	Mn_2^{2+}+Asp_{85}	314.666	312.2494	312.9719	313.0143	313.56328	304.0976	312.5398	312.6499	311.7034

[a]: Each data value represents the mean of at least five molecular simulation experiments;

[b]: --- no related parameter was detected.

Table S4. Correlation between candidate interaction parameters and inhibition data

Pearson correlation analysis data		Combination area (Å^{2})					
		Total	MeAsp^{1}→PP2A	Arg^{2}→PP2A	"Adda"→PP2A	Glu^{4}→PP2A	Mdhb^{5}→PP2A
1 nM	R[a] (*P*[b])	0.473 (0.199)	0.770* (0.015)	0.896** (0.001)	0.678* (0.045)	0.775* (0.014)	0.912** (0.001)
10 nM	R (*P*)	0.571 (0.109)	0.905** (0.001)	0.934** (0.000)	0.785* (0.012)	0.912** (0.001)	0.969** (0.000)
100 nM	R (*P*)	0.651 (0.058)	0.982** (0.000)	0.898** (0.001)	0.855** (0.003)	0.978** (0.000)	0.953** (0.000)
Pearson correlation analysis data		Positive accessible surface area (Å^{2})					
		Total	MeAsp^{1}→PP2A	Arg^{2}→PP2A	"Adda"→PP2A	Glu^{4}→PP2A	Mdhb^{5}→PP2A
1 nM	R (*P*)	0.490 (0.181)	-0.097 (0.805)	-0.468 (0.204)	0.696* (0.037)	0.989** (0.000)	0.541 (0.133)
10 nM	R (*P*)	0.583 (0.100)	0.060 (0.879)	-0.499 (0.171)	0.817** (0.007)	0.963** (0.000)	0.424 (0.255)
100 nM	R (*P*)	0.661 (0.052)	0.193 (0.619)	-0.482 (0.189)	0.891** (0.001)	0.864** (0.003)	0.256 (0.506)
Pearson correlation analysis data		Negative accessible surface area (Å^{2})					
		Total	MeAsp^{1}→PP2A	Arg^{2}→PP2A	"Adda"→PP2A	Glu^{4}→PP2A	Mdhb^{5}→PP2A
1 nM	R (*P*)	0.494 (0.176)	0.439 (0.237)	-0.784* (0.012)	0.492 (0.178)	0.138 (0.723)	0.300 (0.432)
10 nM	R (*P*)	0.416 (0.266)	0.318 (0.404)	-0.763* (0.017)	0.387 (0.303)	0.190 (0.624)	0.271 (0.481)
100 nM	R (*P*)	0.494 (0.176)	0.439 (0.237)	-0.784* (0.012)	0.492 (0.178)	0.138 (0.723)	0.300 (0.432)
Pearson correlation analysis data		Hydrophobic surface area (Å^{2})					
		Total	MeAsp^{1}→PP2A	Arg^{2}→PP2A	"Adda"→PP2A	Glu^{4}→PP2A	Mdhb^{5}→PP2A
1 nM	R (*P*)	0.609 (0.081)	0.676* (0.045)	0.121 (0.757)	0.680* (0.044)	0.058 (0.881)	0.398 (0.289)
10 nM	R (*P*)	0.643 (0.062)	0.723* (0.028)	0.096 (0.806)	0.789* (0.012)	0.082 (0.833)	0.328 (0.389)
100 nM	R (*P*)	0.654 (0.056)	0.721* (0.029)	0.059 (0.880)	0.856** (0.003)	0.083 (0.832)	0.199 (0.608)
Pearson correlation analysis data		Polar surface area (Å^{2})					
		Total	MeAsp^{1}→PP2A	Arg^{2}→PP2A	"Adda"→PP2A	Glu^{4}→PP2A	Mdhb^{5}→PP2A
1 nM	R (*P*)	-0.157 (0.686)	-0.240 (0.533)	-0.716* (0.030)	-0.703* (0.035)	0.092 (0.814)	-0.286 (0.456)
10 nM	R (*P*)	-0.042 (0.915)	-0.019 (0.961)	-0.778* (0.014)	-0.562 (0.116)	0.129 (0.741)	-0.318 (0.404)
100 nM	R (*P*)	0.100 (0.798)	0.197 (0.611)	-0.772* (0.015)	-0.360 (0.341)	0.161 (0.680)	-0.341 (0.369)

Pearson correlation analysis data		Hydrogen bonds (KJ/Mol)							
		Total	MeAsp^1←Arg$_{89}$	Arg^2←Arg$_{214}$	Arg^2→Arg$_{214}$	Arg^2→PRO$_{213}$	"Adda"←Asn$_{117}$	"Adda"←His$_{118}$	Glu^4←Arg$_{89}$
1 nM	R (*P*)	-0.635 (0.066)	0.177 (0.649)	-0.276 (0.472)	-0.088(0.912)	---^{c}	-0.861** (0.003)	-0.932* (0.021)	-0.124 (0.750)
10 nM	R (*P*)	-0.650 (0.058)	-0.022 (0.955)	-0.263 (0.495)	-0.119(0.881)	---	-0.871** (0.002)	-0.956* (0.011)	-0.053 (0.891)
100 nM	R (*P*)	-0.600 (0.088)	-0.214 (0.581)	-0.262 (0.496)	-0.031(0.969)	---	-0.840** (0.005)	-0.967** (0.007)	-0.002 (0.996)

Pearson correlation analysis data		Hydrogen bonds (KJ/Mol)		Ionic bonds (KJ/Mol)					
		Glu^4←TYR$_{265}$	Mdhb^5←Arg$_{89}$	Total	MeAsp^1-Arg$_{89}$	Glu^4-Arg$_{89}$	Glu^4-Mn_2^{2+}	Asp$_{57}$-Mn_1^{2+}	Asp$_{57}$-Mn_2^{2+}
1 nM	R (*P*)	0.469 (0.531)	-0.551 (0.124)	0.258 (0.503)	0.325 (0.393)	0.828** (0.006)	0.141 (0.717)	0.006 (0.987)	-0.679* (0.044)
10 nM	R (*P*)	0.471 (0.529)	-0.684* (0.042)	0.396 (0.291)	0.199 (0.609)	0.722* (0.028)	0.164 (0.674)	-0.206 (0.595)	-0.802** (0.009)
100 nM	R (*P*)	0.479 (0.521)	-0.770* (0.015)	0.474 (0.197)	0.077 (0.844)	0.551 (0.124)	0.161 (0.678)	-0.423 (0.256)	-0.885** (0.001)

Pearson correlation analysis data		Ionic bonds (KJ/Mol)		Metal bonds (KJ/Mol)					
		Asp$_{85}$-Mn_1^{2+}	Asp$_{85}$-Mn_2^{2+}	Total	Glu^4-Mn_2^{2+}	Asp$_{57}$-Mn_1^{2+}	Asp$_{57}$-Mn_2^{2+}	Asp$_{85}$-Mn_1^{2+}	Asp$_{85}$-Mn_2^{2+}
1 nM	R (*P*)	0.571 (0.108)	0.642 (0.062)	0.716* (0.030)	0.313 (0.412)	-0.642 (0.062)	-0.826** (0.006)	0.408 (0.275)	0.346 (0.362)
10 nM	R (*P*)	0.432 (0.245)	0.443 (0.233)	0.721* (0.028)	0.309 (0.419)	-0.615 (0.078)	-0.915** (0.001)	0.330 (0.386)	0.412 (0.271)
100 nM	R (*P*)	0.296 (0.440)	0.317 (0.405)	0.641 (0.063)	0.249 (0.518)	-0.576 (0.104)	-0.953** (0.000)	0.247 (0.521)	0.490 (0.180)

Pearson correlation analysis data		Metal bonds (KJ/Mol)		Active center exposure (Å^2)				
		Asn$_{117}$-Mn_1^{2+}	His$_{241}$-Mn_1^{2+}	Asp$_{57}$ + Asp$_{85}$ + Asn$_{117}$ + His$_{167}$ + His$_{241}$ + Mn_1^{2+}	Asp$_{57}$ + Mn_1^{2+}	Asp$_{85}$ + Mn_1^{2+}	Asn$_{117}$ + Mn_1^{2+}	His$_{167}$ + Mn_1^{2+}
1 nM	R (*P*)	-0.092 (0.815)	0.570 (0.109)	0.250 (0.516)	-0.196 (0.614)	0.538 (0.135)	-0.088 (0.822)	0.522 (0.150)
10 nM	R (*P*)	-0.180 (0.643)	0.635 (0.066)	0.154 (0.693)	-0.424 (0.255)	0.633 (0.068)	-0.074 (0.851)	0.379 (0.314)
100 nM	R (*P*)	-0.256 (0.506)	0.679* (0.045)	0.020 (0.959)	-0.627 (0.071)	0.711* (0.032)	-0.093 (0.813)	0.239 (0.536)

Pearson correlation analysis data		Active center exposure (Å^2)				
		His_{241} + Mn_1^{2+}	Asp_{57} + Asp_{85} + His_{59} + Mn_2^{2+}	Asp_{57} + Mn_2^{2+}	His_{59} + Mn_2^{2+}	Asp_{85} + Mn_2^{2+}
1 nM	R (*P*)	0.412 (0.271)	0.315 (0.409)	-0.673* (0.047)	0.220 (0.570)	0.481 (0.190)
10 nM	R (*P*)	0.226 (0.559)	0.494 (0.177)	-0.758* (0.018)	0.459 (0.214)	0.533 (0.139)
100 nM	R (*P*)	0.011 (0.978)	0.655 (0.055)	-0.786* (0.012)	0.656 (0.055)	0.544 (0.130)

Pearson correlation analysis data		Exposure areas associated with -PO_4 (Å^2)				Combination energy (KJ/Mol)
		Arg_{89} + His_{118} + Arg_{214}	Arg_{89}	His_{118}	Arg_{214}	Toxins→PP2A
1 nM	R (*P*)	0.151 (0.698)	-0.747* (0.021)	-0.552 (0.123)	0.267 (0.487)	-0.553 (0.122)
10 nM	R (*P*)	-0.001 (0.997)	-0.731* (0.025)	-0.671* (0.048)	0.353 (0.437)	-0.503 (0.168)
100 nM	R (*P*)	-0.115 (0.768)	-0.687* (0.041)	-0.726* (0.027)	0.424 (0.255)	-0.413 (0.269)

[a]: R is the Pearson correlation coefficient between candidate interaction parameters and toxin toxicity at different toxin levels；

[b]: *P* is the significant difference of relevant data；

[c]: --- no related parameter was detected;

** indicates significance at the level of 0.01；

* indicates significance at the level of 0.05.
